# *Toxoplasma gondii* seroprevalence in reindeer (*Rangifer tarandus tarandus L.*) in northern Sweden: a cross-sectional study from 2014

**DOI:** 10.1186/s13028-023-00717-7

**Published:** 2023-12-12

**Authors:** Arja Helena Kautto, Abbey Olsen, Camilla Wallander, Ivar Vågsholm

**Affiliations:** 1 Department for Biomedical Sciences and Veterinary Public Health, Swedish University of Agricultural Sciences, Ultuna, Uppsala, Sweden; 2 Section for Animal Welfare and Disease Control, Department of Veterinary and Animal Sciences, University of Copenhagen, Frederiksberg C, Denmark

**Keywords:** Cervid, ELISA, Farmed game, Food safety, Meat safety, Toxoplasmosis, Zoonosis

## Abstract

**Background:**

*Toxoplasma gondii* is a parasitic protozoan that can infect a wide range of warm-blooded animals, including humans. The infection with *T. gondii*, is of particular concern due to its potential impact on human and animal health. In Sweden, semi-domesticated reindeer (*Rangifer tarandus tarandus L.*) is an important species both economically and culturally, but susceptibility to *Toxoplasma* infection and seroprevalence in reindeer herds remain relatively understudied.

**Results:**

A total of 528 reindeer, sampled at two slaughterhouses in Sweden in 2014, were investigated for antibodies to *T. gondii*. Specific antibodies to *T. gondii* were found in 5 of 209 (2.3%) tested adult reindeer and in 6 of 308 (1.9%) tested calves, giving an apparent total prevalence of 2.1% (95% confidence interval 1.1–3.8%). None of four putative risk factors studied (sex, age, type of grazing area, county) were statistically associated with *T. gondii* seroprevalence.

**Conclusions:**

Swedish semi-domesticated reindeer are exposed to *T. gondii* and may harbour infectious tissue cysts. To mitigate the risk of *T. gondii* infection in consumers, reindeer meat should be frozen or cooked thoroughly before consumption. The global climate change may influence the seroprevalence and possible associated risk factors for *T. gondii* in reindeer. To be able to manage the risk and get better advice to the consumers there is a need for further investigations covering the whole spectra of herding conditions for reindeer.

## Background

*Toxoplasma gondii* is a zoonotic protozoan parasite, infective to all warm-blooded animals, with the potential to cause life-threatening disease in individuals with a compromised immune system. Intermediate and definite hosts can both become infected through ingestion of oocysts in contaminated environments, water, fruits, or vegetables or via tissue cysts present in the tissues of another infected animal. Another mode of infection with *T. gondii* is through vertical transmission, where acute infection in pregnant women can lead to congenital toxoplasmosis [1].

Wild and domestic felines are the definitive hosts for *T. gondii*, and when they become infected, they contaminate natural environments, as they can shed millions of oocysts in their faeces [1, 2]. After sporulation, the oocysts can remain infectious for a long time in soil and water [1, 3]. When sporulated oocysts are ingested by a new host, sporozoites are released in the gut and transform into rapidly multiplying tachyzoites. These tachyzoites spread to several tissues, where some differentiate into semi-dormant bradyzoites that are lodged within tissue cysts [1].

Among many other sources, consumption of raw and undercooked meat from infected animals has been epidemiologically associated with infection in humans [4]. In Europe, *T*. *gondii* is ranked as one of the top five foodborne parasitic infections in humans [5]. In Uppsala in east-central Sweden, *T. gondii* seroprevalence in the human population has been found to be 23% [6]. Despite this high seroprevalence in the human population and the potential zoonotic risk, toxoplasmosis in humans is not a notifiable disease in Sweden.

Exposure to *T. gondii* results in a humoral immune response. In several host species, a variety of serological tests can be used to detect anti-*T. gondii* antibodies. In epidemiological studies, immunoglobulin G (IgG) is the most suitable antibody for assessing past exposure to the parasite. Enzyme-linked immunosorbent assays (ELISA) and direct or modified agglutination tests (DAT or MAT) have also been considered suitable for seroepidemiological *T. gondii* studies in many terrestrial animal species [1].

Oocysts in the environment are suggested to be the most likely source of *T. gondii* infection in semi-domesticated reindeer [7] and barren-ground caribou [8–10]. Vertical transmission has also been described in these species [11]. In Sweden, the number of reindeer in commercial slaughter (September-April) has varied between 40,000 and 60,000 per year in the past 10 years and most of the animals slaughtered are calves giving only 1000–1500 tons carcass weight per year [12]. According to an old tradition the reindeer owners in their own households and residents in northern parts of Sweden consume more reindeer meat than the average population.

The seroprevalence of *T. gondii* in reindeer has been found to be low [0–1%] in Fennoscandia [7, 13, 14]. The aim of this study was to determine *T. gondii* seroprevalence in Swedish reindeer by screening animals during commercial slaughter and identify possible risk factors associated with the seroprevalence. The need for further investigations in the reindeer population and for better risk management and advice to consumers was also assessed.

## Methods

### Sampling sites

**Fig. 1:**
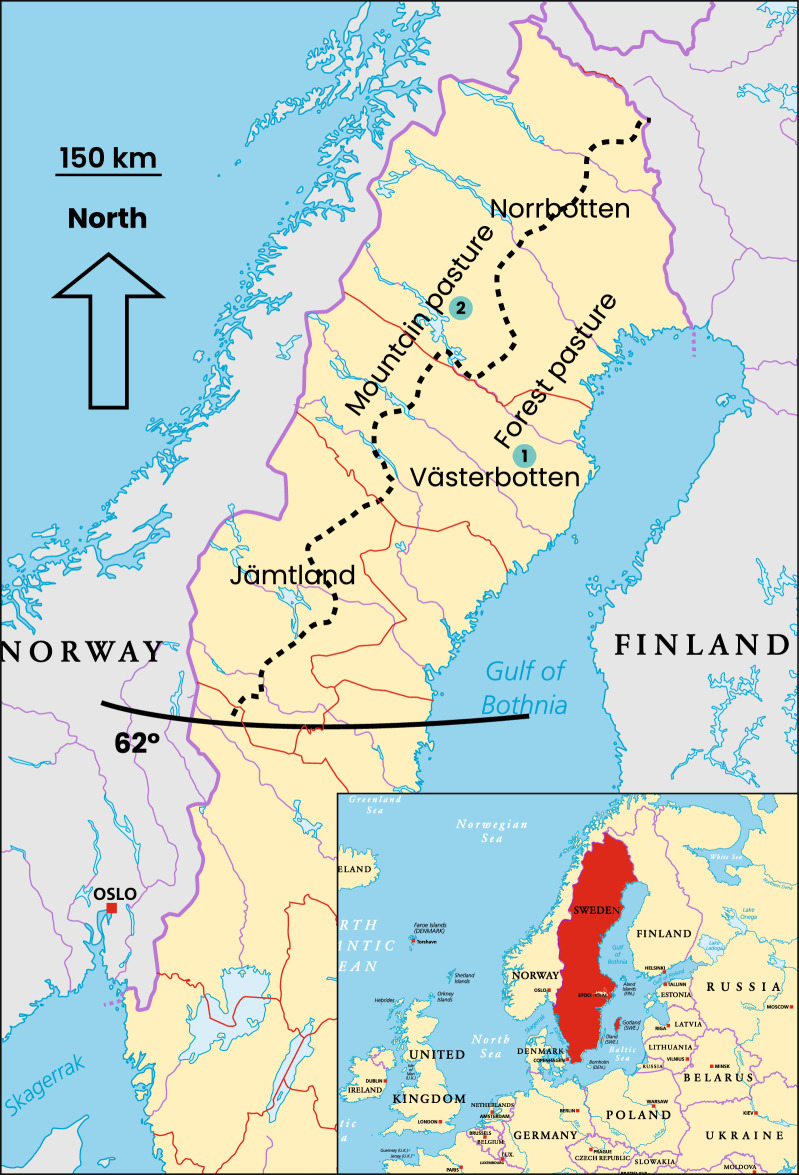
Reindeer herding area in Sweden. Slaughterhouse 1 in Västerbotten and Slaughterhouse 2 in Norrbotten. The southern border of the reindeer herding area (the green line) and the 62° latitude indicated. Red line = the borders between counties, Dotted line roughly showing the border between forest and mountain pasture areas

Sampling took place in 2014 at two commercial slaughterhouses, each slaughtering more than 6000 reindeer annually, controlled by the Swedish Food Agency (Fig. 1). These sites were suitable because they slaughter reindeer from all three counties with herding areas, covering reindeer both from forest and mountain areas. In these counties, reindeer from forest area graze in this area all year-round. However, reindeer from the mountain area have a seasonal pattern in grazing, where they graze in the forest during winter (October to April) and in the mountains from April to September. During the winter season, none of the slaughtered groups were corralled for supplementary or emergency feeding. Furthermore, the duration of pre-slaughter handling was limited to a maximum of two days, including the time for the selection of the slaughter reindeer at the gathering corrals, transport, and the waiting time at the slaughterhouse lairage area.

Food chain information (FCI) was used to categorise each sample according to county and main grazing area (forest/mountain according to the original herding groups). Age (calf/adult) and sex (male/female) of the reindeer were recorded at slaughter.

### Sampling size estimation

Simple random sampling was used to select four sampling weeks (between 15 September and 15 December, with the rutting season 29 September to 20 October excluded 2014) and sampling day per selected week. The number of samples taken per day was a census for the total number of animals slaughtered per day. The sample size was estimated based on a design prevalence of 0.5% i.e., if all samples were negative; the seroprevalence would be at least 0.5% with 95% confidence. The following formula was used to determine the desired sample size:

$$\frac{\mathrm{ln}(1-P)}{\mathrm{ln}(1-Prevalence)}$$

where P = 95% confidence in detecting the disease at a prevalence of 0.5% [15], thus we aimed at sampling 600 reindeer.

### Sampling of reindeer

Blood samples from 528 reindeer were collected in sampling tubes (volume of 10 mL, non-silicon coated, plain without additives, Venoject®, Terumo Europe, Belgium) at the point of bleeding. All clotted samples were centrifuged at 2000 rpm for 5 min (Relative Centrifugal Force, RCF, of 380×*g*) and separated serum samples were without delay frozen in aliquot tubes at − 20 °C.

### Analysis of the samples

A commercial ELISA kit (ID Screen Toxoplasmosis Indirect Multi-species, IDvet Innovative Diagnostics, Montpellier, France) was used to detect *T. gondii*-specific IgG antibodies. This test uses a multi-species conjugate and a purified native surface antigen of *T. gondii* (SAG1/P30) [16]. The test was performed according to the manufacturer’s instructions, where the serum samples were tested at a 1:10 dilution. Percentage sample to positive ratio (S/P%) was calculated using optical density values (OD) of the controls provided by the manufacturer as:

$$\mathrm{S}/\mathrm{P\%}=\frac{\mathrm{OD \,of \,the \,sample}-\mathrm{OD\,of \,the \,negative \,control}}{\mathrm{OD \,of \,the \,positive \,control}-\mathrm{OD \,of \,the \,negative \,control}}*100$$

According to the manufacturer, samples were considered positive at S/P% ≥ 50, inconclusive at S/P% ≥ 40% to < 50% and negative at S/P% < 40%. All samples, including the negative and positive controls from the manufacturer’s kit, were tested in duplicate. In addition to the positive and negative controls provided with the kit, one negative and one positive sample from a previous experimental study on reindeer in Finland was also included and tested in duplicate [17]. The optical density (OD) values for the samples were read at 450 nm (Multiscan FC v. 2^.^ 5, Thermo Fischer Scientific). To evaluate the performance of the ELISA, we evaluated both the intra-assay and inter-assay repeatability. Both the estimates were calculated using raw absorbance values, OD. The intra-plate coefficient of variation (CV%) was calculated as the standard deviation (SD) divided by the mean of the duplicates expressed as percentage. Additionally, for the evaluation of the inter-plate variation, first the means for the two negative and two positive controls were calculated for each plate. These were then used to determine the overall mean, SD, and CV%. The overall CV% was estimated by dividing the pooled SD of the plate means by the mean of these plate means and given in percent. The average CV% from the negative and positive controls was the inter-assay CV% estimate. Similarly, the above-described steps were also applied for estimating inter-assay CV% for the two negative and two positive samples originating from an experimentally infected reindeer. For both intra- and inter-plate, CVs with values < 20% for raw absorbance values, indicated adequate repeatability [18].

Samples were classified as positive or negative, and the odds ratio (OR) for a positive test was estimated for each risk factor using Fischer’s exact test (Software R®) [19]. Since the sensitivity and specificity of the diagnostic test for *T. gondii* in the reindeer population were unknown, we decided to present the proportion of animals that tested seropositive as apparent prevalence (AP) out of the total number of animals tested. In the remainder of this paper, the term seroprevalence refers to AP unless specified otherwise.

## Results

In the performance test for ELISA, the intra-plate repeatability showed a mean CV% value of 3.56% (mean SD = 0.01%), where the CV values of all the samples except four were below 20%. Among these four exceptions, the CV% ranged from 22.2 to 43.04%, where one sample was inconclusive (S/P% = 44.0%), whereas the other samples were negative (S/P% range = 4.1–9.3%). For inter-plate repeatability of the manufacturer’s positive and negative controls, CV% values were below 20%, with the overall mean CV% being 1.39%, representing an average of the pooled CV%s from both positive (CV% = 2.77%) and negative (CV = 2.03%) controls. Similarly, all individual CV% values for inter-plate repeatability of the experimental samples were below 20%, with the overall mean CV% being 5.2%, representing an average of the pooled CV%s from both positive (CV% = 6.09%) and negative (CV = 4.37%) controls.

**Fig. 2:**
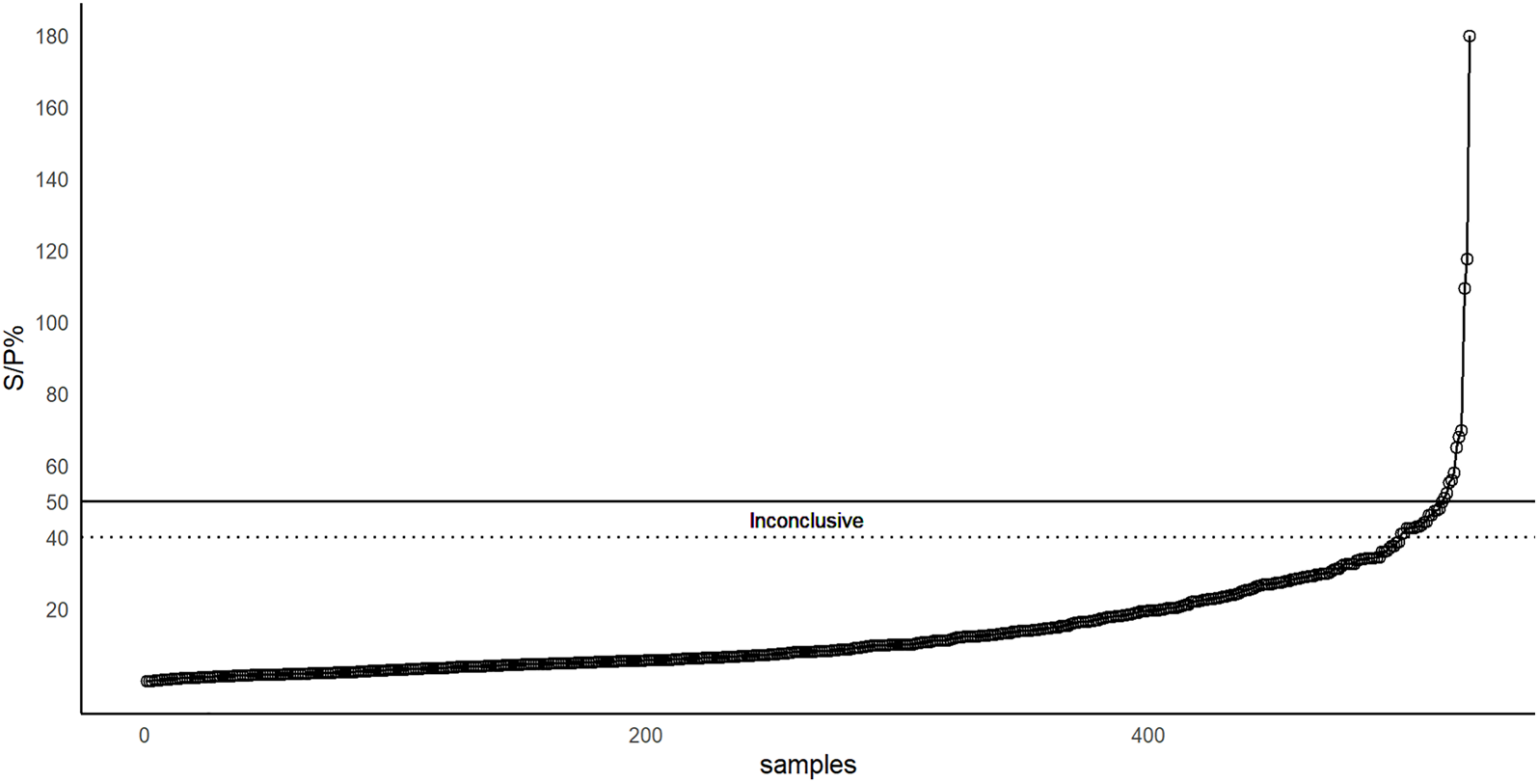
*Toxoplasma gondii*-specific IgG antibodies in Swedish reindeer samples (n = 528), expressed as sample to positive ratio percentage (S/P%) (y-axis). Serum samples were considered to test positive at S/P% ≥ 50% (solid line), inconclusive at S/P% values between ≥ 40% (dotted line) and < 50%, and negative at S/P% < 40%

In total, 11 of the 528 samples tested positive for *T. gondii* antibodies (AP = 2.1%, 95% CI 1.1–3.8%) and 17 samples had inconclusive results (S/P% ≥ 40% to < 50%) (Fig. 2). The seroprevalence was 5.3% (95% CI 3.7–7.6%) when these inconclusive results were included as positives. The seroprevalence was higher in adult reindeer (AP = 2.3%, 95% CI 0.9–5.7%) than in calves (AP = 1.9%, 95% CI 0.8–4.3%), although this difference was not statistically significant (p = 0.7) (Table 1). Furthermore, when inconclusive results were also included, the seroprevalence in calves and adults were 6.4% (95% CI 4.0–9.8%) and 3.7% (95% CI 1.8–7.5%), respectively; however, this difference in the seroprevalence was not statistically significant (p = 0.2).

**Table 1:** Association of *Toxoplasma gondii* seropositivity and putative risk factors (sex, age, forest/mountain area as type of grazing area, geographical region = county) in a study of *T. gondii* in reindeer in Sweden, 2014

Reindeer characteristics		Number of reindeer			Total	Odds ratio (OR)	
		Sero-negative	Seropositive	Inconclusive seronegative		Only conclusive	Including inconclusive
Sex	Female	164	6	3	170	1	1
	Male	353	5	14	358	0.4 (0.1–1.3)	1 (0.4–2.3)
Age	Adult	209	5	3	214	1	1
	Calf	308	6	14	314	0.8 (0.3–2.7)	1.8 (0.8–4.1)
Type of grazing area	Forest	252	6	5	258	1	1
	Mountain	265	5	12	270	0.8 (0.2–2.6)	1.5 (0.7–3.3)
County	Västerbotten	270	6	12	276	1	1
	Norrbotten	247	5	5	252	0.9 (0.3–3.0)	0.6 (0.3–1.3)

Odds ratio with 95% confidence interval (95% CI) is also shown

The sampled reindeer in the study originated from two counties, Norrbotten and Västerbotten, covering both mountain and forest areas (Fig. 1). The risk factor analysis did not reveal any significant associations between the possible risk factors tested (age, sex, main grazing area as mountain vs. forest, county) and *T. gondii* seroprevalence, even when inconclusive seropositive results were included (Table 1).

## Discussion

The seroprevalence of *T. gondii* in the Swedish reindeer was estimated to be 2.1% (Table 1) and comparable with findings in previous studies on reindeer in nearby countries and in Alaska [7, 13, 14, 20]. Due to the low number of seropositive reindeer, the ability to detect associations between possible risk factors such as age, sex, main grazing area and seropositivity as reported in literature for reindeer [7] and other deer species [21] was limited, i.e., the power of detection for possible risk factors in *T. gondii* infection was low.

In this study, the interpretation of the results within the broader context of overall live reindeer population and of *T. gondii* prevalence requires cautious consideration due to various reasons. Firstly, the sampling approach used may have resulted in the exclusion of reindeer from the southern part of the reindeer herding area (Jämtland County), and those from possible feeding corrals. This exclusion could introduce a bias, particularly because *T. gondii* seropositivity is known to be positively associated with degree of domestication and feeding in corrals [7]. Secondly, the reliance on slaughter material as such introduces a selection bias for making inferences about the population of reindeer, as those that die or are withheld from slaughter are not included. Despite these limitations, sampling at slaughter is most relevant for the consumer protection, as we can estimate the consumer exposure to *T. gondii* through reindeer meat. However, considering that the data in this study are from winter 2014, it is possible that various factors, for example pasture conditions, affecting reindeer herding that may have evolved since then may not be fully reflected in our results.

Studies in the past have used MAT to investigate *T. gondii* seroprevalence in wild cervids [13, 14]. However, we used a commercial ELISA test to investigate *T. gondii* seroprevalence in Swedish reindeer, since the results are objective and easier to interpret than those of MAT [22]. In the absence of species-specific conjugates, the ID Screen ELISA kit (Montpellier, France) has been used previously to test for *T. gondii* antibodies in wild cervids [23] and in other wild species [24, 25]. Although the exact reagents present in ID Screen commercial kit were unknown, we assumed that the manufacturer uses some type of non-species-specific antibody binding reagent. We used a 1:10 dilution as a cut-off, as recommended by the manufacturer, and confirmed its validity in reindeer by testing samples taken from a reindeer before and after experimental infection [17]. The S/P% range obtained for these negative and positive sub-samples was 1.0–12.7% and 129.6–167.5%, respectively. Furthermore, as the sensitivity and specificity of the ID Screen ELISA kit for detecting anti-*T. gondii* antibodies in reindeer serum are not yet known, we have reported the apparent seroprevalence without correcting for the diagnostic test error. Hence, the reported seroprevalence values should be interpreted with some caution.

Reindeer are semi-domesticated and graze freely in the vast wilderness north of latitude 62° in northern Sweden, with minimal and only occasional contact with humans (Fig. 1). As a result, there is limited spatial overlap between domestic cats and the reindeer population, which could have contributed to the relatively low seroprevalence of *T. gondii* in reindeer as observed in the two northern counties. Nevertheless, reindeer could become infected with *T. gondii* from domestic cats through consumption of oocyst-contaminated hay or other supplementary feeds, especially during harsh winter conditions. Reindeer grazing in the forest area all year-round are closer to humans, domestic cats as well as lynx, which lives in forest ecosystems. However, our results did not support our initial assumption regarding this risk factor.

A previous study in Russia observed transmission of *T. gondii* via bodily fluids such as saliva and lacrimal fluid within a reindeer herd, suggesting that alternate transmission routes cannot be excluded [26]. There may be a risk of *T. gondii* transmission from wild Eurasian lynx (*Lynx lynx*), since a stable lynx population is present in the Swedish reindeer herding area [27]. Until last two decades, oocysts were not detected in studies analysing faeces samples from Eurasian lynx [28, 29]. However, in 2023, a coprological investigation of free-ranging Eurasian lynx in Switzerland demonstrated presence of oocysts in three out of 176 faeces samples [30]. Thus, oocysts shed by lynx in Sweden into the surrounding pasture areas could pose a risk to reindeer. The Swedish Veterinary Institute does not routinely check for *T. gondii* during lynx necropsies [31]. However, in a serological study conducted in Sweden on 207 samples from Eurasian lynx, the *T. gondii* seroprevalence was found to be 75%. The lynx seroprevalence in the reindeer-herding areas in the north was significantly lower than in the south of Sweden [28]. This north–south gradient is also evident for *T. gondii* seroprevalence in cattle, pigs, and sheep, moose [32, 33] and wild boar in Sweden [34]. A similar geographical trend in seroprevalence has been reported in Finland, specifically in lynx [29], moose and sheep [35]. Higher density of domestic cats in the south (more densely populated areas) is suggested as a possible reason [29]. Other factors such as temperature, humidity, precipitation, snow cover etc. may also influence the distribution and viability of oocysts [36], and local variations in these factors could thus have an impact on *T. gondii* seroprevalence in different populations [37]. In our study the southernmost county was not included and the possible gradient in *Toxoplasma* seropositivity from south of the reindeer herding area up to north could therefore not be studied. Anyhow, even if there is a difference in human population density between Västerbotten and Norrbotten (5 and 3 person per km^2^; respectively) [38] it was not reflected in difference in *Toxoplasma* seropositivity in reindeer in our results. Hence, there is a need for further investigation.

*Toxoplasma gondii* in farmed European deer and wild boar is considered to pose a high risk to safety of meat from those animals [39]. However, due to the lack of evidence that toxoplasmosis can be transmitted to humans through consumption of infected meat from semi-domesticated reindeer, *T. gondii* is not currently considered to pose a high risk in reindeer meat safety. It is important to note that relatively few studies have investigated *T. gondii* seroprevalence in semi-domesticated reindeer [40], meaning that it cannot definitively be asserted that the risk is absent or low. Given that reindeer meat is widely consumed in Sweden, the potential risk from infected animals cannot be dismissed, but the extent to which reindeer meat contributes to human toxoplasmosis remains unknown. The results in this study indicated a low seroprevalence of *T. gondii* in reindeer in Sweden. Finding antibodies gives a historical perspective—the reindeer have been infected with *Toxoplasma*, while not necessarily currently presenting a risk for transmission of *T. gondii*. Hence, a study looking for the presence of tissue cysts in both serologically positive and negative reindeer is needed, as the relationship between anti-*T. gondii* IgG and presence of tissue cysts seems to vary between animal species and does not provide a clear indication of the risk to consumers [41]. When planning future studies of risk factors for *T. gondii* seropositivity in reindeer, the low seroprevalence found in this study should be taken into account when calculating the sample size needed.

Our results indicate that although only a small percentage of Swedish reindeer may have been exposed to *T. gondii*, they could potentially carry tissue cysts that can infect humans. Tissue cysts have been shown to be resilient, with viable tissue cysts being isolated from decomposing tissues several days after the death of the animal. The parasite can survive even at low temperatures of 4–6 ℃ for up to 2 months [1]. This suggests that reindeer meat infected with *T. gondii* could pose a risk of transmission to other animals. From a zoonotic perspective, to mitigate the risk of infection in humans, it is recommended that reindeer meat should be frozen at least overnight at − 12.4 °C, which usually renders tissue cysts non-viable [42] and even colder temperature and longer time can make tissue cysts non-infective [32, 42]. However, a study in Russia found that *T. gondii* remained viable in reindeer meat stored at between − 11 °C and − 56 °C for up to 28 days [43]. This suggests that the effectiveness of freezing in completely eliminating *T. gondii* may vary under different conditions, leaving some uncertainty regarding the absolute mitigation of risk through freezing alone. Furthermore, the low-fat content in reindeer meat could possibly result in further reductions in tissue cyst viability during processing (curing, drying), as reported for ham [44]. However, it should be noted that food preparation methods such as microwave cooking, salting, curing, and pickling have been found to be ineffective in eliminating tissue cysts [1]. To prevent infection, it is recommended that reindeer meat be cooked to 67 °C or higher, to render the tissue cysts non-infectious [45].

Ungulates, among many wildlife species, may be affected directly or indirectly by climate change, for example through changes in habitat or food sources [46]. In northern and Western Europe, climate change has been associated with higher temperature and increased rainfall at higher altitudes [47]. This can lead to extended vegetation periods [48], potentially prolonging the survival of *T. gondii* oocysts in the environmental [47], and thereby increasing the risk of exposure in reindeer through contaminated water or forage. Seroprevalence of *T. gondii* has been shown to be higher following wetter summers in polar bears (*Ursus maritimus*), while living for a longer time on the land because of the loss of sea ice [49]. Similarly, changes in reindeer behaviour due to altered habitats could intensify both the infection risk in these animals and the associated zoonotic threat [50]. The strong spatial variation in precipitation patterns over the reindeer herding area in Fennoscandia [50], suggests differing environmental conditions for various reindeer populations. Therefore, to be able to better manage the risk and give advice to the consumers, there is a need for further investigations covering different types of reindeer husbandry, including different kind of feeding conditions and possible changes in these patterns in future. Additionally, warmer climates might also expand the range of definitive hosts like felids, which shed the oocysts. As a climate-sensitive infection, global climate change could profoundly influence *T. gondii* prevalence by reshaping local environments and anthropogenic factors [51], highlighting the importance of deeper research into *T. gondii* prevalence in reindeer.

## Conclusions

The Swedish reindeer population is apparently exposed to *T. gondii* parasites during grazing and may harbour infectious tissue cysts. Reindeer meat is widely consumed in Sweden and, if eaten raw or undercooked, it could be a potential source of *T. gondii* infection in humans. Individuals who consume reindeer meat should ensure that the meat is safe by using cooking and freezing methods known to effectively neutralise *T. gondii*. The global climate change may influence the future seroprevalence and associated risk factors for *T. gondii* in reindeer. Hence, there is an obvious need for further investigation of *T. gondii* in reindeer.

## Data Availability

The dataset analysed during the current study is available from the corresponding author upon request.

## Abbreviations

AP: Apparent prevalence
DAT: Direct Agglutination Test
ELISA: Enzyme-linked immunosorbent assay
FCI: Food chain information
IgG: Immunoglobulin type G
MAT: Modified agglutination test
OD: Optical density
OR: Odds ratio
RCF: Relative Centrifugal Force
SD: Standard deviation
S/P: Sample/positive ratio
